# The effectiveness of enhanced evidence-based care for depressive disorders: a meta-analysis of randomized controlled trials

**DOI:** 10.1038/s41398-021-01638-7

**Published:** 2021-10-16

**Authors:** Le Xiao, Han Qi, Wei Zheng, Yu-Tao Xiang, Thomas J. Carmody, Taryn L. Mayes, Madhukar H. Trivedi, Gang Wang

**Affiliations:** 1 The National Clinical Research Center for Mental Disorders & Beijing Key Laboratory of Mental Disorders, Beijing Anding Hospital & the Advanced Innovation Center for Human Brain Protection, Capital Medical University, Beijing, China; 2 Department of Psychiatry, The Affiliated Brain Hospital of Guangzhou Medical University (Guangzhou Huiai Hospital), Guangzhou, China; 3 Unit of Psychiatry, Institute of Translational Medicine, Faculty of Health Sciences, University of Macau, Macao SAR, China; 4 Center for Cognition and Brain Sciences, University of Macau, Macao SAR, China; 5 Institute of Advanced Studies in Humanities and Social Sciences, University of Macau, Macao SAR, China; 6 Department of Population and Data Sciences, University of Texas Southwestern Medical Center, Dallas, Texas USA; 7 Department of Psychiatry, University of Texas Southwestern Medical Center, Dallas, Texas USA

**Keywords:** Depression, Diseases

## Abstract

Several care models have been developed to improve treatment for depression, all of which provide “enhanced” evidence-based care (EEC). The essential component of these approaches is Measurement-Based Care (MBC). Specifically, Collaborative Care (CC), and Algorithm-guided Treatment (AGT), and Integrated Care (IC) all use varying forms of rigorous MBC assessment, care management, and/or treatment algorithms as key instruments to optimize treatment delivery and outcomes for depression. This meta-analysis systematically examined the effectiveness of EEC versus usual care for depressive disorders based on cluster-randomized studies or randomized controlled trials (RCTs). PubMed, the Cochrane Library, and PsycInfo, EMBASE, up to January 6th, 2020 were searched for this meta-analysis. The electronic search was supplemented by a manual search. Standardized mean difference (SMD), risk ratio (RR), and their 95% confidence intervals (CIs) were calculated and analyzed. A total of 29 studies with 15,255 participants were analyzed. EEC showed better effectiveness with the pooled RR for response of 1.30 (95%CI: 1.13–1.50, *I*^2^ = 81.9%, *P* < 0.001, 18 studies), remission of 1.35 (95%CI: 1.11–1.64, *I*^2^ = 85.5%, *P* < 0.001, 18 studies) and symptom reduction with a pooled SMD of −0.42 (95%CI: −0.61–(−0.23), *I*^2^ = 94.3%, *P* < 0.001, 19 studies). All-cause discontinuations were similar between EEC and usual care with the pooled RR of 1.08 (95%CI: 0.94–1.23, *I*^2^ = 68.0%, *P* = 0.303, 27 studies). This meta-analysis supported EEC as an evidence-based framework to improve the treatment outcome of depressive disorders.

**Review registration:** PROSPERO: CRD42020163668

## Introduction

Evidence-based care is the use of the best available evidence together with clinical judgement, as well as patient preferences, to make healthcare decisions. Over the past 50 years, several psychotherapies and pharmacological interventions have demonstrated efficacy and safety in the treatment of depressive disorders. Yet remission rates remain low, with only about one-in-three individuals achieving remission in acute treatment trials. In fact, Pence and colleagues estimate that only about 6% of individuals with depression in primary care achieve remission [1]. Clearly, efforts are needed to improve outcomes for depression treatment.

Several care models have been developed to improve treatment for depression, all of which provide “enhanced” evidence-based care (EEC). The essential component of all of these approaches is Measurement-Based Care (MBC). MBC in psychiatry is defined as the use of validated clinical measurement instruments to objectify the assessment, treatment, and clinical outcomes, including efficacy, safety, tolerability, functioning, and quality of life, in patients with psychiatric disorders [2]. The concept of MBC was derived from the stepwise treatment algorithm by Trivedi et al [3].

Rigorous assessment and treatment algorithms of MBC are regarded as key instruments to optimize treatment delivery and outcomes for MDD [4, 5]. These explicit treatment protocols aim at a predefined treatment goal (e.g., remission or response). Treatment algorithms provide strategies (which treatments to use), tactics (how to implement the treatments), treatment steps (in what order to implement the different treatments), standardized evaluation instruments, critical decision points and standardized medical decisions based on preset “if-then-rules” [6]. Simply put, MBC is “the routine measurement of symptoms and side effects at each treatment visit and the use of a treatment manual describing when and how to modify medication doses based on these measures” [3]. MBC uses patient-reported rating scales in conjunction with evidence-based clinical practice guidelines to provide an objective assessment of patient progress over time to guide a more effective plan of care [7].

Other enhanced evidence-based treatment strategies include Collaborative Care (CC), Integrated Care (IC), and Algorithm-Guided Treatment (AGT), all of which use varying forms of rigorous MBC assessment, care management, and/or treatment algorithms as key instruments to optimize treatment delivery and outcomes for depression. CC and IC integrate assessment, care management, low-intensity psychotherapeutic interventions and antidepressant medication in conformity with evidence-based guidelines [8–10], and also include procedural elements of MBC, and have demonstrated a significant improvement in depressive symptoms. AGT which is standardized stepwise drug treatment regimen (SSTR), has also demonstrated efficacy for MDD [6, 11]. The broad concept of MBC as the systematic evaluation of patient symptoms to inform behavioral health treatment includes the components of CC and IC [12]. Because these strategies all use components of MBC to enhance evidence-based care, we refer to these strategies (MBC, AGT, CC, and IC) as EEC.

Previous studies showed that AGT, MBC, CC, and IC for MDD were superior to treatment as usual (TAU) [6, 11, 13–18]. In addition, a few previous systematic reviews and meta-analyses have been published, but separately reviewed AGT and CC programs and outcomes [10] [19–21], or overviewed MBC for adolescent depression [22]. However, we do not know the overall effectiveness of these four strategies (referred to as EEC hereafter) using measurement, coordinated, and guideline-based care for MDD. To date, no systematic review or meta-analysis has been published to explore the overall effectiveness of such enhanced strategies for adults with MDD. Thus, we performed this meta-analysis of RCTs to systematically evaluate the effectiveness of EEC in depressive disorders.

## Materials and methods

This meta-analysis was conducted following the Preferred Reporting Items for Systematic Reviews and Meta-Analyses (PRISMA) statement [23], with the registration number of No. CRD42020163668.

### Searching strategy

PubMed, the Cochrane Library, PsycInfo, and EMBASE databases were systematically and independently searched by two researchers (LX, HQ) from the inception dates (Pubmed:1966, the Cochrane Library: 1995, EMBASE: 1974, PsycInfo: 1872) up to January 6th, 2020. Search terms included: (depressi*) AND (“measurement-based” OR “algorithm” OR “collaborative care” OR “integrated care”). Relevant reviews were also screened manually for additional studies.

### Study selection and study criteria

The same two researchers independently screened the titles and abstracts of relevant publications and then read the full texts for eligibility. The publications were reported in English. Inclusion criteria were made based on the ***PICOS*** acronym: ***P***articipants: patients with major depressive disorder or other depressive disorders according to study-defined diagnostic criteria. ***I***ntervention: AGT, MBC, CC, or IC. Comparison: usual care, treatments as usual or standard treatments. ***O***utcomes: Primary outcome measure included response and remission defined with any standardized rating scales, such as the Hamilton Depression Rating Scale (HAMD), Montgomery-Asberg Depression Rating Scale (MADRS), The Symptom Checklist (SCL) or the Beck Depression Inventory (BDI) for depression. If the above-mentioned response and remission were not reported, then study-defined response and remission were included for analyses (Supplementary Table 1). Key secondary outcomes included 1) the reduction of total scores measured by any rating scales between baseline and endpoint, 2) all-cause discontinuation during the study period. Study design: RCTs and cluster-randomized studies. Exclusion criteria: 1) comorbid physical diseases; 2) special populations, such as children and adolescents; 3) no information of treatment step or strategy.

### Data extraction

The same two researchers independently conducted data extraction. Any disagreement in the procedures was resolved by a discussion or by consulting senior researchers (YTX and WZ).

Relevant study and participant characteristics were recorded using a preprepared data collection sheet. Additional information was obtained by contacting first or corresponding authors if necessary. For studies with cross-over or sequential parallel design, only data in the first randomized study phase prior to treatment change was extracted.

### Quality assessment

Risk of bias (ROB) was assessed using the Cochrane Risk of Bias tool [24], and the JADAD scale [25]. The JADAD scale scored from 0 to 5, with the total score of ≥3 considered “high quality” [25]. The evidence level and the strength of recommendations of the meta-analysis were measured using the grading of recommendations assessment, development, and evaluation (GRADE) system as recommended by the Cochrane Collaboration [26, 27]. Any disagreement in the procedure was resolved by discussion between the authors (H.Q. and L.X.).

### Statistics

Due to different participant and study characteristics across studies, the random-effects model was used in all analyses. Intent-to-treat (ITT) data were preferred over observed cases data. Standardized mean difference (SMD) and risk ratio (RR) with 95% confidential intervals (CIs) were adopted for continuous and categorical outcomes, respectively. The I [2] statistic was used to assess heterogeneity between studies [28], and significant heterogeneity was defined as I [2] statistics of > 50% [28].

Potential sources of heterogeneity for primary and secondary outcomes were examined by subgroup analyses based on the following variables: (i) PHQ-9 vs. SCL vs. others; (ii) trial duration (months): ≥6 vs. <6 (6 months was chosen using the weighted mean split of trial duration); (iii) Open label vs. single-blind; (iv) MBC/AGT vs. CC. The moderating effects of JADAD score, mean age, and gender proportion on the results were assessed using meta-regression analysis. Publication bias was tested with funnel plots and the Egger test [29]. Significant level was set as *P* < 0.05 (two-tailed). Data were analyzed using STATA Version 15.1.

## Results

### Literature search

**Fig. 1:**
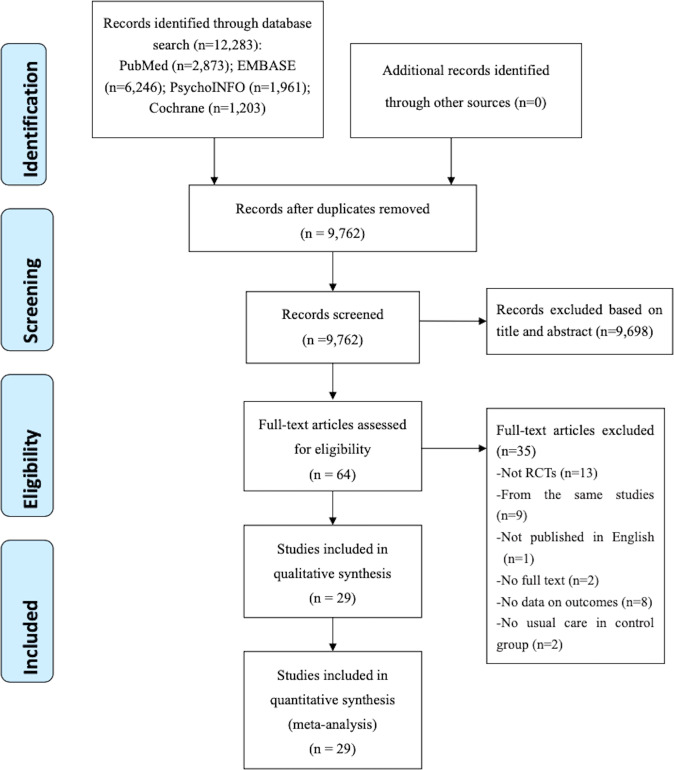
PRISMA flow diagram of database search results and article selection.

Altogether, 12,283 relevant publications were identified in the literature search. After removing duplicates, 9762 were assessed by titles and abstracts. Of these, the full texts of 65 papers were read for eligibility. Eventually, 29 studies were included for analysis (Fig. 1).

### Study Characteristics

Study and participant characteristics of the 29 RCTs with a total of 15,255 participants are summarized in Table 1. The mean age of participants was 52.18 (Standard deviation ranged from 6.03 to 17.10) years, and the mean proportion of males was 37.7%. Thirteen studies were conducted in the US, and others were done in Germany (4), Spain (1), UK (6), China (1), Japan (1), Netherlands (2), and Italy (1). Twenty-two studies were based on multi-center and 7 were single-center design. Twenty-one studies used CC, the remaining used MBC or AGT, and no IC studies were eligible for inclusion criteria.

**Table 1:** Patients and characteristics of the included studies.

Author / Year	Intervention	Number of centers	Blinding	Analyses	Duration (mean)	Number of patients (ITT)	Mean age (yrs)	Male (%)	Scale	JADAD score
Adli, 2017 [6]	AGT	10	No	LOCF	20w	429	44.2	36.83	HAMD-21	3
Alexopoulos, 2009 [35]	CC	20	No	OC	24 m	599	NR	28.38	HAMD-24	2
Aragonès, 2014 [40]	CC	20	Single	OC	12 m	338	47	20.12	PHQ-9	2
Bauer, 2009 [11]	MBC	1	No	LOCF	12w	148	48.2	40.54	BRMS	3
Bosanquet, 2018 [31]	CC	4	No	NR	18 m	415	71.9	82.89	PHQ-9	3
Camacho, 2018 [41]	CC	36	Single	NR	24 m	387	58.5	62.02	SCL-D13	3
Chaney, 2011 [33]	CC	10	No	LOCF	7 m	546	64.2	96.15	PHQ-9	2
Chew-Graham, 2007 [30]	CC	43	Single	OC	16w	105	75.5	27.62	SCL-20	3
Ell, 2010 [42]	CC	2	No	NR	18 m	387	NR	17.83	PHQ-9	3
Finley, 2003 [43]	CC	1	No	NR	6 m	125	54.28	15.20	BIDS	2
Fortney, 2007 [44]	CC	7	Single	OC	12 m	395	59.2	91.65	SCL-20	2
Gilbody, 2017 [32]	CC	32	Single	NR	12 m	705	77	42.27	PHQ-9	3
Guo, 2015 [13]	MBC	1	Single	LOCF	24w	120	41.1	35.83	HAMD-17	3
Harter, 2018 [17]	SCM	49	NR	LOCF	12 m	737	42.1	26.59	PHQ-9	3
Huijbregts, 2013 [45]	CC	18	Single	OC	12 m	150	48.67	NR	PHQ-9	3
Hunkeler, 2006 [34]	CC	18	Single	OC	12 m	1801	71.2	35.00	SCL-20	2
Katon, 1999 [8]	CC	4	Single	NR	6 m	228	47.8	27.70	SCL-20	3
Katon, 2004 [46]	CC	9	Single	NR	12 m	329	58.35	35.00	SCL-90	3
Lagomasino, 2017 [36]	CC	3	No	OC	16w	400	49.6	16.75	PHQ-9	3
Menchetti, 2013 [37]	CC	16	Single	ITT	12 m	227	51.8	23.79	PHQ-9	3
Richards, 2013 [47]	CC	51	Single	OC	12 m	581	44.8	28.06	PHQ-9	3
Richards, 2008 [48]	CC	1	Single	OC	3 m	114	42.47	22.81	PHQ-9	3
Ricken, 2011 [49]	MBC	1	No	OC	20w	148	48.2	40.54	BRMS	3
Simon, 2000 [50]	CC	5	Single	OC	6 m	613	46.6	17.46	HSCD-20	3
Solberg, 2015 [51]	CC	75	No	NR	6 m	2348	44.4	27.30	PHQ-9	2
Unützer, 2002 [16]	CC	18	Single	NR	12 m	1801	71.2	35.15	SCL-20	3
Vlasveld, 2012 [52]	MBC	1	No	NR	12 m	126	42.63	46.03	PHQ-9	3
Yeung, 2012 [14]	AGT	74	Single	ITT	6 m	642	46	33.64	PHQ-9	2
Yoshino, 2009 [15]	AGT	1	No	NR	54 m	210	48.9	50.95	CGI	2

*AGT* algorithm-guided treatment, *BIDS* Brief Inventory for Depressive Symptoms, *CC* collaborative care, *HSCD-20* 20 item depression scale from the Hopkins symptom checklist, *ITT* intent-to treat, *MBC* measurement-based care, *NR* not report, *OC* observed cases, *SCM* stepped care model, which was treated as MBC in this study.

### Quality assessment

The mean JADAD score of included studies was 2.63, with 20 (68.7%) studies rated as “high quality (≥3)” (Table 1). Twenty-one (72.4%) studies described the method of randomization, but none used double blind randomization. The Cochrane risk of bias of the 29 studies is presented in Supplementary Table 2. Twenty-one studies had low risk for random sequence generation, 16 had unclear risks for allocation concealment, and nine had high risks for the blinding of participants and personnel. GRADE evaluation found that all the primary and secondary outcomes had a moderate level of recommendation because of serious inconsistency between studies (Supplementary Table 3).

### The effects of EEC on MDD

**Fig. 2:**
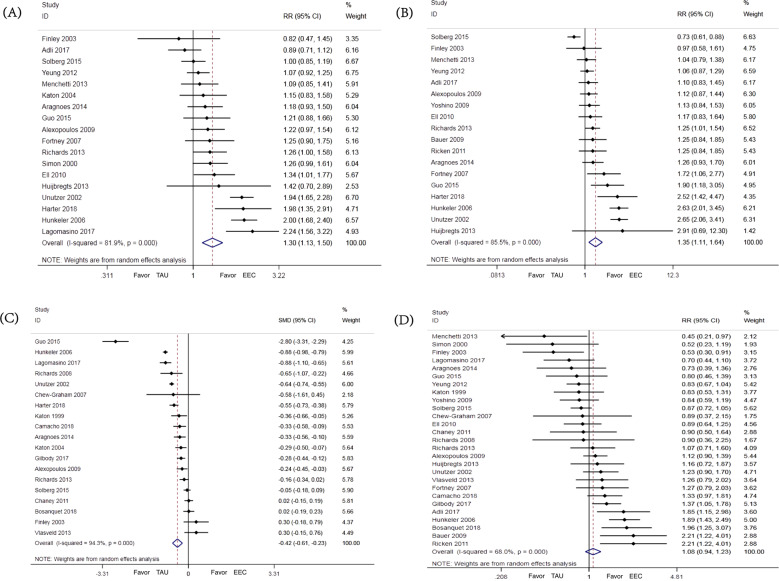
Effects of EEC on MDD. **A** Response; **B** Remission; **C** Improvement of depressive symptoms; **D** Discontinuation.

The results showed that compared to controls, EEC could effectively improve response rate with the pooled RR of 1.30 (95%CI: 1.13–1.50, *I*^2^ = 81.9%, *P* < 0.001, 18 studies), and remission rate with the RR of 1.35 (95%CI: 1.11–1.64, *I*^2^ = 85.5%, *P* < 0.001, 18 studies) (Figs. 2A and B). Compared to controls, the scores of standardized scales decreased significantly more during the study period in the EEC group with a SMD of −0.42 (95%CI: −0.61–(−0.23), *I*^2^ = 94.3%, *P* < 0.001, 19 studies) (Fig. 2C). No group difference in discontinuation rate was found with the pooled RR of 1.08 (95%CI: 0.94–1.23, *I*^2^ = 68.0%, *P* = 0.303, 27 studies) (Fig. 2D).

### Subgroup and meta-regression analyses

Subgroup analyses were performed to explore the sources of heterogeneity for response rate, remission rate and changes of scale scores during the study period (Table 2). With respect of response rate comparing EEC with TAU, studies using the PHQ-9 (RR: 1.28, 95%CI: 1.10–1.50, *I*^2^ = 69.6%, *P* = 0.002) and SCL (RR: 1.60, 95%CI: 1.24–2.05, *I*^2^ = 78.8%, *P* < 0.001) showed that the EEC group had a higher pooled response rate, but a significant difference was not observed in the studies using other scales (*P* = 0.244). The single-blind, duration > 6 months, and CC studies supported that the response rate of EEC was greater than TAU, but the studies using MBC/AGT (*n* = 5) did not show a significant difference.

**Table 2:** Subgroup analyses of efficacy and safety of EEC in the treatment of depressive disorders.

	Subgroups	Categories (Number of Studies)	Sample size	RRs/SMDs	95% Confidence Interval (%) [Lower, Upper]	*I*^*2*^ (%)	*P* within subgroup
Response rate	Scales	PHQ-9 (9)	4794	1.28	[1.10, 1.50]	69.6	0.002
		SCL (4)	3962	1.60	[1.24, 2.05]	78.8	<0.001
		Others (5)	1300	1.10	[0.94 1.30]	40.1	0.244
	Blind	Open label (7)	3823	1.26	[0.99, 1.61]	81.1	0.057
		Single-blind (11)	6233	1.33	[1.12, 1.57]	81.3	0.001
	Duration (weeks)^#^	≤u months (7)	3493	1.15	[0.96, 1.37]	73.1	0.140
		> 6 months (11)	6563	1.41	[1.20, 1.66]	76.2	<0.001
	Intervention	MBC + AGT (5)	2241	1.19	[0.97, 1.47]	71.6	0.100
		CC (13)	7815	1.34	[1.13, 1.59]	82.2	0.001
Remission rate	Scales	PHQ-9 (8)	4461	1.16	[0.93, 1.44]	76.5	0.183
		SCL (3)	3636	2.47	[2.00, 3.03]	26.6	<0.001
		Others (7)	1414	1.18	[1.04, 1.34]	0.0	0.013
	Blind	Open label (9)	3996	1.13	[0.93, 1.37]	69.0	0.231
		Single blind (9)	5515	1.60	[1.20, 2.14]	87.5	0.001
	Duration (weeks)^#^	≤u months (7)	3064	1.09	[0.88, 1.36]	71.6	0.424
		>6 months (11)	6447	1.54	[1.20, 2.00]	84.7	0.001
	Intervention	MBC + AGT (7)	2355	1.28	[1.06, 1.54]	51.9	0.010
		CC (11)	7156	1.37	[1.12, 1.64]	90.4	0.036
Changes of scale scores	Scales	PHQ-9 (10)	5750	−0.26	[−0.45, −0.07]	88.1	0.007
		SCL (6)	4408	−0.53	[−0.75, −0.31]	88.2	<0.001
		Others (3)	558	−0.90	[−2.47, 0.66]	97.9	0.256
	Blind	Open label (7)	3975	−0.22	[−0.47, 0.04]	91.4	0.099
		single-blind (12)	6741	−0.55	[−0.79, −0.31]	93.9	<0.001
	Duration (weeks)^#^	≤u months (7)	3060	−0.71	[−1.28, −0.13]	95.7	0.016
		>6 months (12)	7656	−0.30	[−0.50, −0.10]	93.7	0.003
	Intervention	MBC + AGT (2)	1499	−1.66	[−3.87, 0.54]	98.5	0.139
		CC (17)	9217	−0.30	[−0.48, −0.12]	93.1	0.001

*AGT* Algorithm-guided treatment, *CC* collaborative care, *MBC* measure-based care, *PHQ-9* Patient Health Questionnaire-9 item, *SCL-20* 20 item of Symptom Checklist.

Note: *P* < 0.05 was considered statistically significant. ^#^using median splitting method.

With respect of remission rate, studies using the SCL (RR: 2.47, 95%CI: 2.00–3.03, *I*^2^ = 26.6%, *P* < 0.001) and other scales (RR: 1.18, 95%CI: 1.04–1.34, *I*^2^ = 0%, *P* = 0.013) supported that EEC is superior to TAU, which was not observed in the studies using PHQ-9 (*P* = 0.183). Similar to response rate, single-blind, duration >6 months studies favored EEC to TAU. Both the MBC/AGT (*n* = 7) and CC (*n* = 11) studies showed that the enhanced intervention had higher remission rate than TAU (*P* < 0.05).

With respect of the improvement of symptoms, studies using the PHQ-9 (SMD: −0.26, 95%CI: −0.45 − (−0.07), *I*^2^ = 88.1%, *P* = 0.007) and the SCL (SMD: −0.53, 95%CI: −0.75 – (−0.31), *I*^2^ = 88.2%, *P* < 0.001) showed that EEC had greater symptom reduction. Single-blind studies supported EEC had a greater reduction in symptoms than TAU, which was not observed in open-label studies. In both long- and short- term duration of studies, EEC was superior to TAU in the reduction of scale scores. With regard to the specific EEC, CC led to greater symptoms improvement compared to TAU (SMD: −0.30, 95% CI: −0.48 − (−0.12), *I*^2^ = 93.1%, *P* = 0.001), however, this was not shown in the studies using MBC/AGT (*P* = 0.139).

Meta-regression analysis found that older mean age was positively associated with higher remission rate (*β* = 0.04 *P* = 0.012). JADAD scores and proportion of males were not significantly associated with response rate, remission rate, and changes of scale scores.

### Publication bias, sensitivity analysis

Egger’s test for response rate (*t* = 1.25, *P* = 0.229), remission rate (*t* = 0.55, *P* = 0.590), and changes of scale scores (*t* = −1.18, *P* = 0.254) indicated no publication bias. Funnel plots of the response and remission rate are shown in Supplementary Figure 1 and 2. Sensitivity analysis of pooled RR for response and remission rate is shown in Supplementary Figure 3 and 4, and there was no significant change in primary results when included studies were removed one by one.

## Discussion

Enhanced evidence-based care strategies, such as MBC, CC, and AGT have demonstrated improved outcomes over treatment as usual. This is the largest meta-analysis of 29 RCTs with a total of 15,255 patients targeting the effectiveness of EEC in the treatment of depressive disorders. Of the 29 studies, 18 studies reported the number or rate of response and remission, and pooled RR of response and remission rate were 1.30 and 1.35, respectively, showing that EEC is superior to usual care. Nineteen studies reported the change in the depressive symptoms, and EEC showed a significant decrease of depressive symptoms compared with usual care with small effect size (SMD = −0.42). These findings supported the effectiveness of EEC strategies for depressive disorders compared to usual care, which are consistent with the findings of the previous meta-analyses of CC [19] and MBC in depressed adolescents [22]. This study highlights the value of EEC for management of depressive disorders.

Subgroup analyses found that response, remission and improvement of symptoms were moderated by rating scales, study design (single-blind or open-label), study duration and intervention type when comparing EEC with TAU. Meta-regression showed that older age was positively associated with the remission rate. In this meta-analysis, six studies included have examined the effectiveness of EEC in older people [16, 30–34], and all of these studies used PHQ-9 or SCL to assess the efficacy outcome. The duration in five of the geriatric studies was longer than 6 months [16, 31–34], and four out of six studies were single-blinded [16, 30, 32, 34]. Given the overlap in these factors, this may explain the finding that older people benefit most from EEC for depression. Alternatively, another explanation may be that some studies enrolled patients with subthreshold or mild depression [15, 17, 32] and some studies partly included minor depression or dysthymia [11, 16, 17, 34–37], which could enhance the remission rate at endpoint. The rating scales and definition of response and remission could also affect the overall outcomes. Therefore, the effect of associated factors on the treatment outcome of EEC should be further examined.

All-cause discontinuation did not differ significantly between EEC and usual care, which demonstrated the good acceptability and feasibility of EEC in clinical practice. One concern, of course, is whether patients will agree to regular monitoring of symptoms, which is the key component of MBC. In fact, routine self-assessment is not the burden of the patients, by contrast, monitoring their symptoms and side effects can help them understand the nature of their depression and the complexity of its treatment. All these factors are beneficial in improving the acceptability of the illness management [38].

The following limitations of this meta-analysis should be acknowledged. First, the number of eligible studies of this meta-analysis for response (18 RCTs), remission (18 RCT), and symptoms improvement (19 RCTs) were relatively small, which increases the type II error [39]. Second, significant heterogeneity of the results regarding response (*I*^2^ = 81.9%), remission (*I*^2^ = 85.5%), and symptoms improvement (*I*^2^ = 94.3%) was found. Finally, the subgroup and meta-regression analyses were only conducted between EEC as a whole and TAU, the efficacy of the different types of interventions (MBC, AGT, CC) was not compared due to limited number of studies (*n* = 8) using MBC/AGT.

In conclusion, this meta-analysis confirmed the effectiveness and acceptability of enhanced evidence-based care, such as measurement-based care, algorithm-guided treatment, and collaborative care, in the management of depressed patients. As an evidence-based framework, EEC could also reduce variability in psychiatric treatment [7]. Moreover, EEC can be utilized across a diverse range of settings, disorders and treatment, so it is conceptualized as a transdiagnostic and transtheoretical practice [12]. Therefore, we recommend some forms of EEC in clinical practice and psychiatry residency training in the future.

## Supplementary information

Supplementary material
