# Supplementary material for: The effectiveness of enhanced evidence-based care for depressive disorders: a meta-analysis of randomized controlled trials
Source: Transl Psychiatry. 2021 Oct 16;11:531. doi: 10.1038/s41398-021-01638-7 (PMC8520525; doi:10.1038/s41398-021-01638-7)
Supplement: Supplementary file 1 — Supplementary material [file 41398_2021_1638_MOESM1_ESM.docx]

**Supplementary materials**

**Supplementary Table 1.** Characteristics of the included studies

| **Author / Year** | **Diagnosis & Tool** | **Response** | **Remission** | **Improvement of depression** |
| --- | --- | --- | --- | --- |
| Adli, 2017 | Major depressive episode by ICD-10 | Reduction of HAMD-21≥50% | HAMD-21 ≤9 | ND |
| Alexopoulos, 2009 | Major depression by DSM-IV, or minor depression defined as 3-4 depressive symptoms, HAMD-24≥10 | Reduction of HAMD-24≥50% | HAMD-24 ≤9 | Reduction of HAMD-24 |
| Aragonès, 2014 | Major depression by DSM-IV and PHQ-9 ≥10 | Reduction of PHQ-9 ≥50% | PHQ-9 <5 | Reduction of PHQ-9 |
| Bauer, 2009 | Major depressive episode, dysthymia, longer depressive reaction and bipolar depression by ICD-10 | ND | BRMS ≤7 | ND |
| Bosanquet, 2018 | Major depressive disorder by MINI | ND | ND | Reduction of PHQ-9 |
| Camacho, 2018 | PHQ-9≥10 | ND | ND | Reduction of SCL-D13 |
| Chaney, 2011 | Major depression by PHQ-9≥10 | ND | ND | Reduction of PHQ-9 |
| Chew-Graham, 2007 | Major depressive disorder by DSM-IV, Geriatric Depression Scale ≥5 | ND | ND | Reduction of HSCL |
| Ell, 2010 | PHQ-9≥10 | Reduction of SCL-20 score ≥50% | SCL-20<0.5 | ND |
| Finley, 2003 | Depression by clinical judgement | Reduction of BIDS≥50% | BIDS<9 | Reduction of BIDS |
| Fortney, 2007 | PHQ-9 ≥12 | Reduction of SCL-20 score≥50% | SCL-20<0.5 | ND |
| Gilbody, 2017 | subthreshold depression by DSM-IV | ND | ND | Reduction of PHQ-9 |
| Guo, 2015 | Major depression by DSM-IV | Reduction of HAMD-17≥50% | HAMD-17 ≤7 | Reduction of HAMD-17 |
| Harter, 2018 | PHQ-9≥5 | Reduction of PHQ-9 ≥50% | PHQ-9 <5 | Reduction of PHQ-9 |
| Huijbregts, 2013 | Major depressive disorder by MINI, PHQ-9 ≥10 | Reduction of PHQ-9 ≥50% | PHQ-9 <5 | ND |
| Hunkeler, 2006 | Major depression,or dysthymia | Reduction of SCL-20 ≥50% | SCL-20 < 0.5 | Reduction of SCL-20 |
| Katon, 1999 | 4 or more persistent major depressive symptoms by physicians or HSCL depression items ≥ 1.5 | ND | ND | Reduction of SCL-20 |
| Katon, 2004 | PHQ-9 ≥10 | Reduction of SCL-20 ≥40% | ND | Reduction of SCL-20 |
| Lagomasino, 2017 | Major depressive disorder or dysthymia by PHQ-9 | Reduction of PHQ-9≥50% | ND | Reduction of PHQ-9 |
| Menchetti, 2013 | Minor or major depression by DSM-IV | Reduction of PHQ-9≥50% | PHQ-9 <5 | ND |
| Richards, 2013 | Depressive episode by ICD-10 | Reduction of PHQ-9≥50% | PHQ-9 ≤9 | Reduction of PHQ-9 |
| Richards, 2008 | Major depression by a SCID depression score ≥5 | ND | ND | Reduction of PHQ-9 |
| Ricken, 2011 | Depressive disorder by ICD-10 with an indication for antidepressive pharmacotherapy | ND | BRMS score ≤7 | ND |
| Simon, 2000 | Depression by 20 item depression scale from HSCL | Reduction of depression scores on HSCL ≥50% | ND | ND |
| Solberg, 2015 | Depression using the PHQ-9 measure of severity | Reduction of PHQ-9≥50% | PHQ-9 < 5 | Reduction of PHQ-9 |
| Unützer 2002 | Major depression or dysthymia by SCID | Reduction of SCL-20 ≥50% | SCL-20<0.5 | Reduction of SCL-20 |
| Vlasveld, 2012 | Major depressive disorder by PHQ-9 | Reduction of PHQ-9≥50% (NR) | PHQ-9 <5 (NR) | Reduction of PHQ-9 |
| Yeung, 2012 | Major depressive disorder by their physician | Reduction of PHQ-9≥50% | PHQ-9 <5 | ND |
| Yoshino, 2009 | Mild or moderate MDD by DSM-IV | ND | CGI =1 over 4 weeks | ND |

**Abbreviations:** BIDS =Brief Inventory for Depressive Symptoms; CGI =Clinical Global Impression; DMS-IV =Diagnostic and Statistical Manual of Mental Disorders, Fourth Edition; HAMD = Hamilton Depression Rating Scale; ICD-10 =International Classification Of Diseases, 10th revision; HSCL = Hopkins Symptom Checklist; ND =Not Done; NR=Not Reported; PHQ-9 =Patient Health Questionnaire-9 item; SCID =Standard Clinical Interview for DSM-IV; SCL-20 = 20-item of Symptom Checklist

**Supplementary Table 2. Cochrane Risk of bias**

| Author (Year) | Random sequence generation (selection bias) | Allocation concealment (selection bias) | Blinding of participants and personnel (performance bias) | Blinding of outcome assessment (detection bias) | Incomplete outcome data (attrition bias) | Selective reporting (reporting bias) | Other bias |
| --- | --- | --- | --- | --- | --- | --- | --- |
|  |  |  |  |  |  |  |  |
| Adli (2017) | low risk | low risk | high risk | low risk | low risk | low risk | low risk |
| Bauer (2009) | low risk | low risk | high risk | high risk | high risk | low risk | low risk |
| Ell (2010) | low risk | low risk | high risk | low risk | low risk | low risk | low risk |
| Finley (2003) | unclear risk | low risk | low risk | unclear risk | high risk | low risk | low risk |
| Guo (2015) | low risk | unclear risk | low risk | low risk | low risk | low risk | low risk |
| Katon (2004) | low risk | unclear risk | low risk | low risk | low risk | low risk | low risk |
| Solberg (2015) | unclear risk | unclear risk | low risk | low risk | low risk | low risk | low risk |
| Unützer (2002) | low risk | low risk | high risk | low risk | low risk | low risk | low risk |
| Yoshino (2009) | unclear risk | unclear risk | low risk | high risk | low risk | low risk | low risk |
| Alexopoulos (2009) | unclear risk | unclear risk | high risk | low risk | low risk | low risk | low risk |
| Aragonès (2014) | unclear risk | unclear risk | low risk | low risk | low risk | low risk | low risk |
| Chaney (2011) | low risk | unclear risk | unclear risk | unclear risk | low risk | low risk | low risk |
| Chew-Graham (2007) | low risk | low risk | low risk | low risk | low risk | low risk | low risk |
| Fortney (2007) | unclear risk | unclear risk | unclear risk | unclear risk | low risk | low risk | low risk |
| Hunkeler (2006) | unclear risk | unclear risk | low risk | unclear risk | high risk | low risk | low risk |
| Gilbody (2017) | low risk | low risk | low risk | low risk | low risk | low risk | low risk |
| Huijbregts (2013) | low risk | low risk | low risk | unclear risk | low risk | low risk | low risk |
| Lagomasino (2017) | low risk | unclear risk | unclear risk | unclear risk | low risk | low risk | low risk |
| Menchetti (2013) | low risk | unclear risk | high risk | high risk | low risk | low risk | low risk |
| Richards (2008) | low risk | low risk | low risk | low risk | low risk | low risk | low risk |
| Richards (2013) | low risk | low risk | low risk | low risk | low risk | low risk | low risk |
| Ricken (2011) | low risk | unclear risk | high risk | high risk | low risk | low risk | low risk |
| Simon (2000) | low risk | unclear risk | low risk | low risk | low risk | low risk | low risk |
| Vlasveld (2013) | low risk | low risk | low risk | low risk | low risk | low risk | low risk |
| Yeung (2012) | unclear risk | unclear risk | low risk | low risk | low risk | low risk | low risk |
| Bosanquet (2018) | low risk | low risk | high risk | high risk | low risk | low risk | low risk |
| Camacho (2018) | low risk | low risk | low risk | low risk | low risk | low risk | low risk |
| Katon (1999) | low risk | unclear risk | low risk | low risk | low risk | low risk | low risk |
| Harter (2018) | low risk | unclear risk | high risk | high risk | low risk | low risk | low risk |

**Supplementary Table 3. GRADE analysis of the efficacy and safety of EEC for depressive disorders.**

| **Primary/secondary outcome** | **Studies (N)** | **Risk of bias** | **Inconsistency** | **Indirectness** | **Imprecision** | **Publication bias** | **Large effect** | **Overall quality of evidence ^a^** |
| --- | --- | --- | --- | --- | --- | --- | --- | --- |
| Response rate | 18 (10,056) | No ^c^ | Serious ^b^ | No | No | Undetected | No | +/-/+/+/; Moderate |
| Remission rate | 18 (9,511) | No ^c^ | Serious ^b^ | No | No | Undetected | No | +/-/+/+/; Moderate |
| Symptom reduction | 19 (10,716) | No ^c^ | Serious ^b^ | No | No | Undetected | No | +/-/+/+/; Moderate |
| All cause discontinuation | 27 (14,130) | No ^c^ | Serious ^b^ | No | No | Undetected | No | +/-/+/+/; Moderate |

Note: GRADE=grading of recommendations assessment, development, and evaluation.

a: High quality=further research is very unlikely to change our confidence in the estimate of effect. Moderate quality=further research is likely to have an important impact on our confidence in the estimate of effect and may change the estimate. Low quality=further research is very likely to have an important impact on our confidence in the estimate of effect and is likely to change the estimate. Very low quality=we are very uncertain about the estimate.

b: Meta-analytic results presented a serious inconsistency when I^2^ values were greater than 50% or P<0.1 in the Q statistics.

c: Meta-analytic studies only mentioned random allocation without describing the method.

d: RR>2 or RR<0.5.


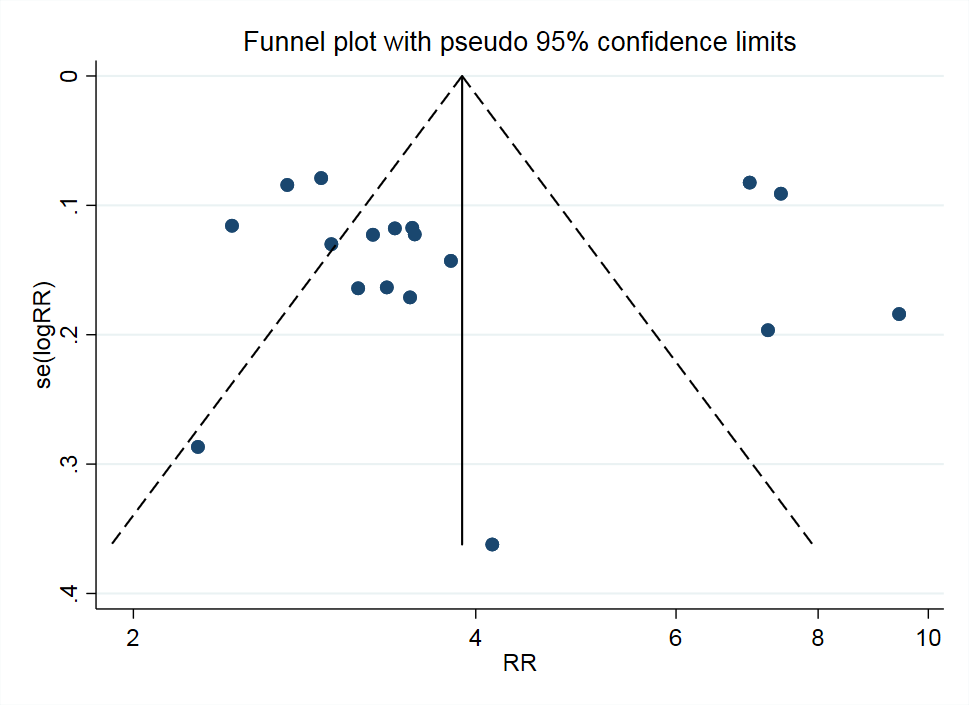


**Supplementary Figure 1.** Funnel plot of the response rate of EEC for depressive disorders


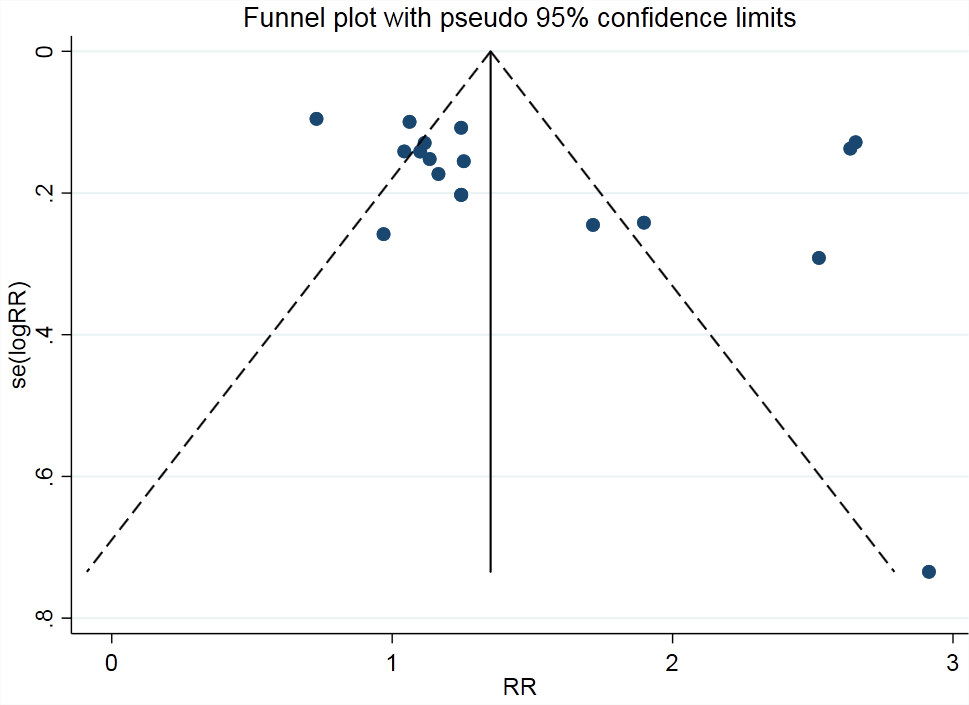
**Supplementary Figure 2.** Funnel plot of the remission rate of EEC for depressive disorders


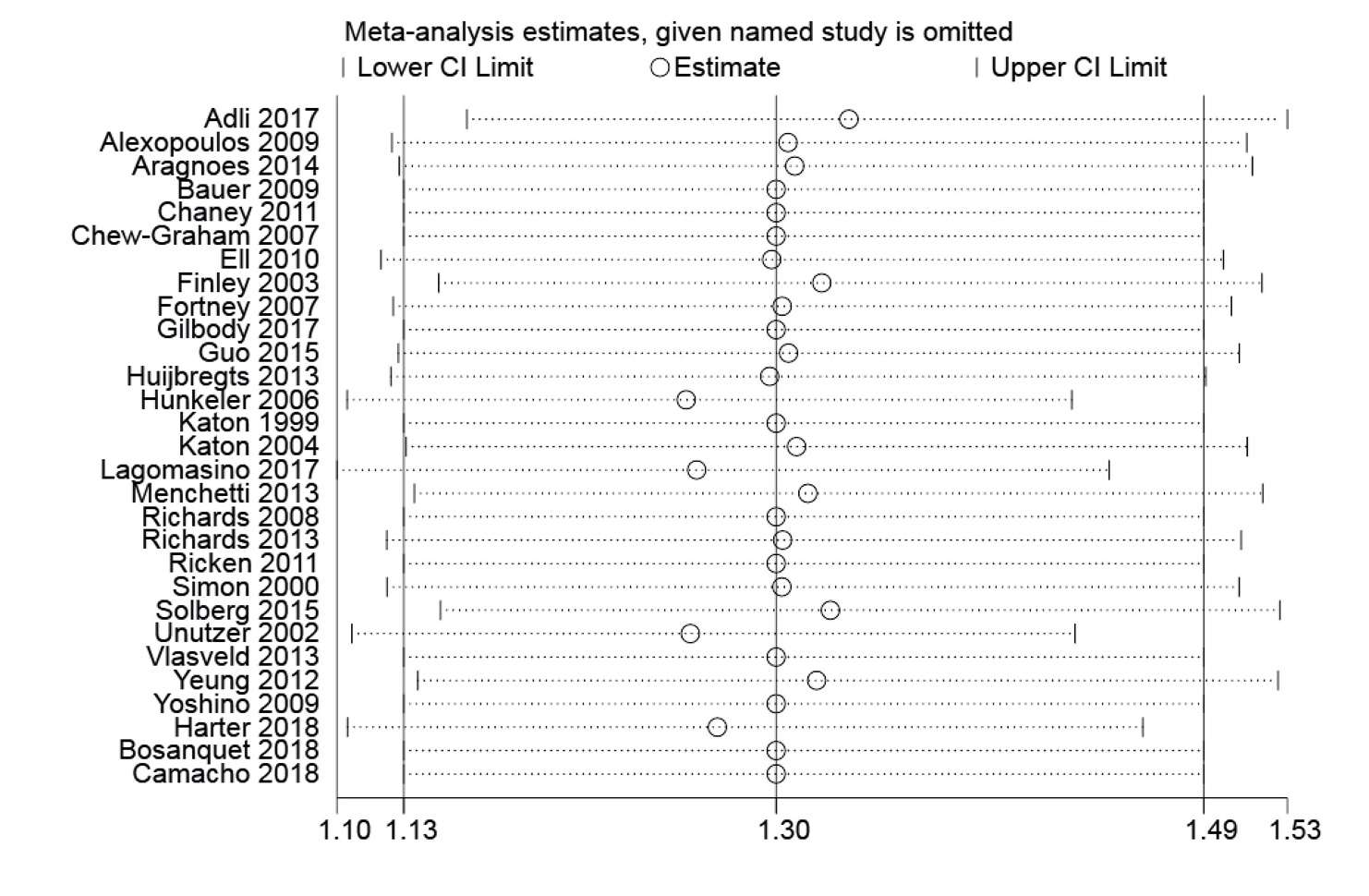


**Supplementary Figure 3.** Sensitivity analysis of pooled RR for the response rate of EEC vs TAU


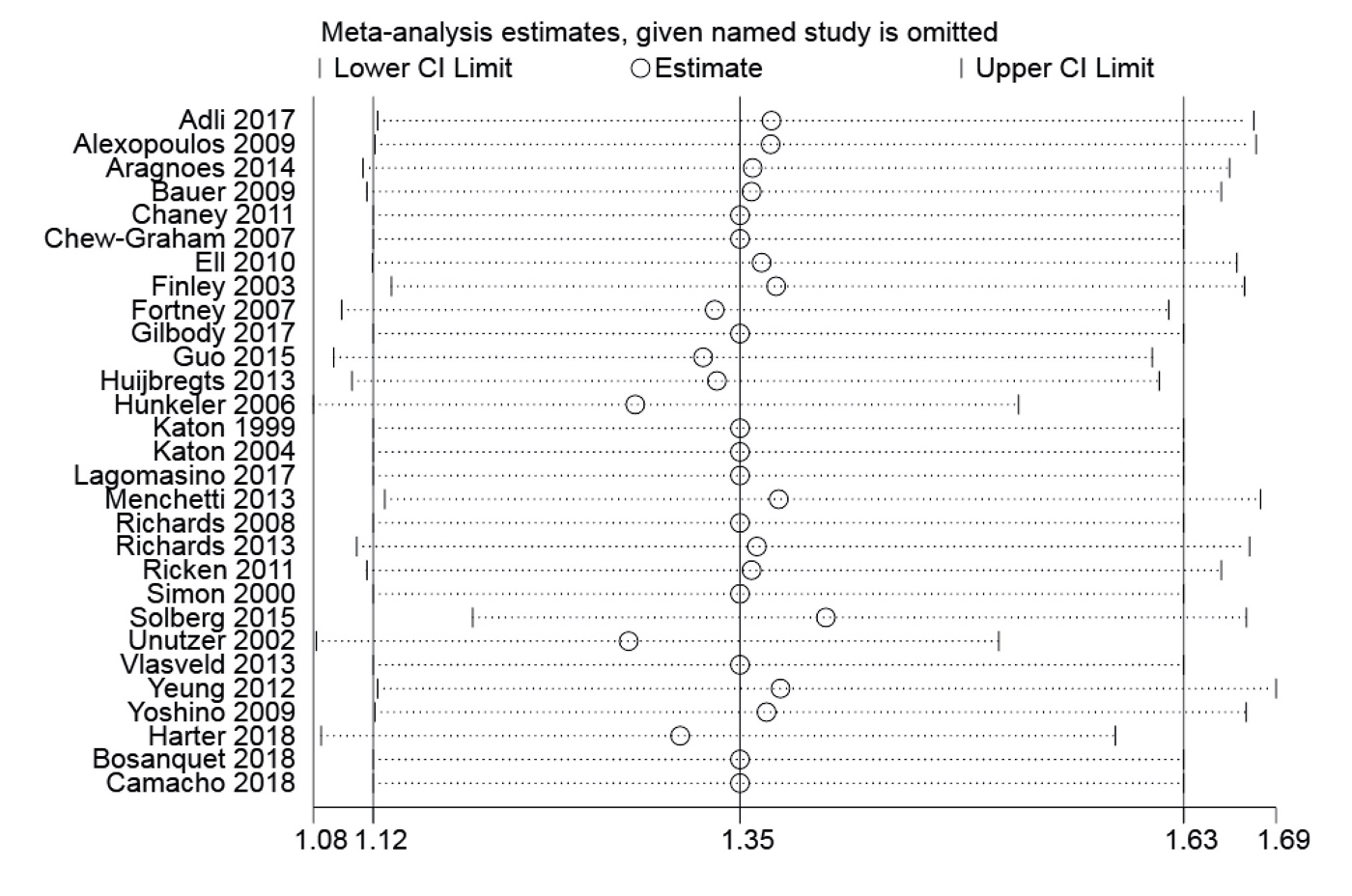
 **Supplementary Figure 4.** Sensitivity analysis of pooled RR for the remission rate of EEC vs TAU
